# Sustainable Management of Photovoltaic Waste Through Recycling and Material Use in the Construction Industry

**DOI:** 10.3390/ma18020284

**Published:** 2025-01-10

**Authors:** Sandra Bulińska, Agnieszka Sujak, Michał Pyzalski

**Affiliations:** 1 Department of Biosystems Engineering, Faculty of Environmental and Mechanical Engineering, Poznań University of Life Sciences, Wojska Polskiego 50, 60-627 Poznan, Poland; sandra.bulinska@up.poznan.pl; 2 Faculty of Materials Science and Ceramics, AGH University of Krakow, Al. Mickiewicza 30, 30-059 Kracow, Poland; michal.pyzalski@agh.edu.pl

**Keywords:** solar energy waste, photovoltaic cells, post-operational waste, construction industry, cementitious materials, construction materials, cement composites

## Abstract

The rapid expansion of photovoltaic (PV) technology as a source of renewable energy has resulted in a significant increase in PV panel waste, creating environmental and economic challenges. A promising strategy to address these challenges is the reuse of glass waste from decommissioned PV panels as a component of cementitious materials. This review explores the potential of integrating glass waste from PV panels into cementitious materials, focusing on its impact on their mechanical, thermal, and durability properties. This analysis includes various methods of processing PV glass waste, such as crushing and grinding, to obtain the desired particle size for cementitious applications. It goes on to analyze how advances in cementitious materials can facilitate the incorporation of PV glass waste, helping to improve properties such as compressive strength, workability, and setting time. In addition, this review makes a detailed analysis of the long-term sustainability and environmental benefits of PV glass waste, highlighting its potential to reduce the carbon footprint of cementitious materials. Incorporating PV glass waste can improve certain properties of cementitious materials, resulting in increased durability and improved thermal insulation, while contributing to waste reduction and resource conservation. This review highlights the importance of developing standardized recycling methods and integration processes and identifies areas for further research to optimize the use of PV glass waste in cement formulations. Ultimately, the sustainable integration of PV glass panel waste into cementitious materials is a viable approach to promote green building practices and support a circular economy in the construction industry.

## 1. Introduction

The production and consumption of electricity is one of the main scientific issues to eliminate progressive climate change. Global energy demand is increasing every year. This trend is due to the intensification of anthropogenic impacts and the dynamic development of new technologies. These technologies, including heating and cooling systems, increasingly rely on electricity [1]. Ubiquitous climate change, including global warming and increasing energy consumption, forces the energy sector to implement clean, low-emission energy sources [2]. With the depletion of fossil fuels and the need to reduce environmental emissions, the ideal solution is to replace conventional energy sources with renewable ones. One alternative is to obtain electricity directly from the sun’s rays through the photovoltaic phenomenon, which generates a voltage by absorbing ionizing radiation [3]. Inside the cell at the p–n junction, incident solar radiation causes the excitation of electrons and the generation of excitons, which are then split into free charge carriers, i.e., electrons and holes [4]. The enormous potential of energy extracted from the sun is related to the direct conversion of solar radiation into electrical energy. Of the annual energy emitted by the sun, about 40 percent, estimated at roughly 15^14^ MWh, arrives; the remainder is reflected from the high layers of the atmosphere.

The use of photovoltaic panels has made it possible to obtain renewable, clean energy [5] and quiet operation of the device [6]. The popularity of renewable energy sources, including solar power, is playing a key role in the energy transition worldwide. Photovoltaics are widely used, not least due to their lower carbon emissions of 23–81 g CO_2_-eq/kWh, compared to fossil fuels (400–1000 g CO_2_-eq/kWh) [7]. CO_2_-eq (carbon dioxide equivalent) is a metric that quantifies the total greenhouse gas emissions, including gases like methane (CH_4_) and nitrous oxide (N_2_O), expressed as an equivalent amount of CO_2_ based on their global warming potential (GWP). For solar photovoltaic (PV) systems, the CO_2_-eq savings can be estimated by comparing the energy they produce with the emissions avoided from conventional energy sources, which vary by region (e.g., 700–800 g CO_2_-eq/kWh in coal-reliant countries like Poland). However, it is essential to consider the carbon footprint of PV panel production, which adds approximately 20–50 g CO_2_-eq/kWh over their lifetime [8].

The number of photovoltaic installations is increasing yearly for both private and industrial use. In 2010, the global installation capacity was 39 GW [9], and solar energy supply surpassed 100 GW for the first time in 2017. In 2020, supply was as high as 139 GW, but this capacity had already reached 1.3 TW last year. Such a rapid development of solar technologies is possible due to cost reductions of solar energy conversion technologies, increasing public environmental awareness, and political support at the local and global levels. An average annual growth rate for PV of 7.7% by 2040 is forecasted [10].

It is important to remember that photovoltaic panels, like any device, have a limited lifetime of around 25 years. The first generation of panels is approaching the end of their life cycle, meaning they need to be disposed of, refurbished, or recycled with minimal environmental impact. Panel waste is estimated to reach 2 million tons by 2038 [11] and by 2050, more than 70 million tons of solar waste will be produced globally, with more than 15 million tons coming from the European Union countries alone [12]. The amount of waste will continue to increase as there is an increasing focus on decarbonizing the energy sector and meeting climate targets.

Effective management of end-of-life waste from photovoltaic panels is crucial, as nearly 90% of such waste currently ends up in landfills. This practice poses significant environmental risks due to the leaching of harmful substances. Addressing the challenges of panel waste management can be integrated into a global strategy aimed at establishing a sustainable, long-term, and cost-effective system, supported by the development of new industrial sectors [12]. In response to this challenge, the European Commission introduced Directive 2012/19/EU, which classifies photovoltaic panels as electrical and electronic equipment and obliges producers, distributors, and sellers to collect and recycle them, including financing, reporting, and administration [13]. Managing photovoltaic panel waste requires cooperation in several areas of technology, regulation, and public awareness. Effective recycling and waste management are key to the sustainability of renewable energy and minimizing environmental impacts.

## 2. PV Construction

Photovoltaics is a technology that generates electricity as direct current (DC), with the generated power typically measured in watts (W) or kilowatts (kW). The energy is produced when photons from sunlight illuminate semiconductors. This process occurs as light falls on a solar cell, exciting the electrons in the semiconductor material and resulting in an electric current [14]. Solar energy is a temporary energy resource whose availability depends on the time of day and atmospheric conditions. Meanwhile, the demand for electricity at any given time shows variability depending on the specific application and geographical location. This results in only a certain proportion of solar energy used in electricity production [15,16]. Initially, photovoltaic technologies were used in space, but they can now be found wherever sunlight is available and there is an electricity demand [17]. Solar energy has become the third most used renewable source worldwide, after hydropower and wind power. This is primarily due to its low CO_2_ emissions, which significantly contribute to combating global warming.

Generating electricity from fossil fuels results in carbon dioxide emissions of 400 to 1000 g CO_2_-eq/kWh [18]. Solar power plants convert the energy of sunlight into electricity through the photovoltaic effect. In this process, photons from solar radiation transfer their energy to electrons in the atoms of a semiconductor material, typically silicon, within the crystal lattice. This energy excites the electrons, causing them to break free from their atomic bonds and generate an electric current under the influence of an internal electric field within the photovoltaic cell. The internal electric field of the p–n junction directs the charge carriers, allowing current to flow through the electrical circuit before electrons recombine with electron holes. This process occurs in photovoltaic panel systems used for individual and industrial purposes [19]. The number of panels in a system depends mainly on the power required and the available surface area for the utility.

Photovoltaic farms contain many components, such as photovoltaic cells, electrical connections, mechanical fixings, support structures, cabling, safety components, controls, and additional components [20]. Due to the mechanical forces acting on the support structures, materials such as steel and aluminum are used, which are strong and resistant to deformation [20,21].

**Figure 1:**
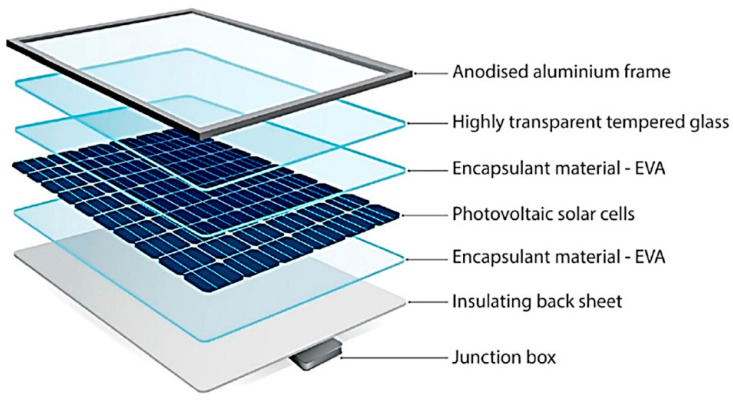
PV module components (reprinted with the permission of GSES) [24].

Photovoltaic panels are designed to maintain performance and durability for many years. They consist of multiple protective and structural layers (Figure 1). The key components of a photovoltaic panel are as follows:

1. Photovoltaic Cells: These semiconductor elements are capable of generating current through the photovoltaic phenomenon. They are made of p–n junctions and silicon cells. Two types of silicon are used:
  - Monocrystalline cells are characterized by high efficiency and are made from a single silicon crystal, produced by the Czochralski process [22].
  - Polycrystalline cells are less expensive but less efficient and made from multiple silicon crystals.
2. Protective Layers: These layers protect the photovoltaic cells using toughened glass for weather protection and ethylene vinyl acetate (EVA) film, which laminates the cells and holds them in place for transparency. The back sheet provides additional protection against moisture and dust.
3. Aluminum Frame: This frame protects the panel edges from mechanical damage and corrosion, ensures rigidity, and facilitates installation.
4. Multi-contact Connectors and Cabling: These components electrically connect the panels and other system components, protecting them from the elements. The cables are made of tin-plated copper with double insulation and are UV radiation-resistant.
5. Anti-reflective Coating: This coating is made of titanium oxide (TiOx) or silicon nitride (SiNx) increases light absorption by reducing its reflection [23].
6. Bypass Diodes: These diodes allow current to flow, bypassing damaged cells to prevent power loss and overheating.

This design maximizes the use of solar energy with minimal losses, contributing to the durability and efficiency of photovoltaic systems.

Among solar cells, it is the crystalline cells made of silicon that utilize intensive phosphorus doping in the n+ type regions that are responsible for electron generation [25]. These heavily doped layers are located on the front surface of p-type silicon, doped with boron to effectively accept the generated electrons. This creates the p–n junction, which is a key element in the operation of a photovoltaic cell.

At the back of the silicon substrate is a back surface field (BSF) region, which minimizes losses due to the recombination of minority carriers, i.e., electrons generated by sunlight [26]. BSF areas are created by firing an aluminum paste applied to the back surface using screen printing and thermal annealing [27].

The electrons generated from the silicon orbitals and in the diffusion regions are collected by silver electrodes (contacts) placed on the front and back surfaces of the cell. The front contact has a ‘comb’ structure, consisting of thin lines connected to a larger collector rail. The rear contact is made up of strips of silver, which are connected by soldered copper wires to the front busbar of the adjacent cell to ensure continuity of current flow.

These contacts are formed by silver paste screen-printing [28], and firing is performed in the same process as the BSF areas are formed. In addition, to increase the light absorption efficiency, the front surface of the cell is coated with an anti-reflective coating (ARC). The front silver contact is inserted into the ARC layer using a baking process, which ensures direct contact with the n+ silicon layer.

Screen-printing technology [29] of silver front contacts with burn-in through the ARC layer is a commonly used method for mass production. Alternative technologies, such as the use of boron-doped BSF or contacts coated with a nickel-copper layer, are used by a small number of manufacturers.

Typical efficiencies of commercial silicon solar cells with standard structures are around 16–18% for monocrystalline substrates and 15–17% for polycrystalline substrates, with cell thicknesses of 160 to 240 μm. These cells are assembled into modules of multiple cells soldered and laminated onto a glass panel, where ethylene vinyl acetate (EVA) is used as the encapsulant. The efficiency of modules made up of such cells is typically about 2% lower than that of a single cell, giving an approximate efficiency at the 12–15% level [23].

## 3. Types of Photovoltaic Panels

Photovoltaic cells, as unitary elements in solar energy conversion systems [30], can be classified based on production technology into thin-film and thick-film technologies [19]. They can be divided into three main groups: silicon panels, thin-film panels, and third-generation panels [1]. Thin-film panels include cells made from copper indium gallium selenide (CIGS) and cadmium telluride (CdTe). Thin-film solar cells constitute the second generation of semiconductor solar cells, made from materials such as CdTe and CIGS [7]. Third-generation panels represent modern technologies, such as perovskite cells, alternative CIGS solutions, and concentrating photovoltaic cells.

However, the majority of installations worldwide rely on silicon cells, which account for about 85% of the energy produced. Silicon cells can be divided into two types: monocrystalline (mono-Si) and polycrystalline (multi-Si). These widely used technologies consist of cells containing crystalline silicon and are characterized by high efficiency and reliability [31]. 

Crystalline silicon cells are categorized based on their structure:

- Monocrystalline (Mono-Si) Cells: These are made from a single, continuous silicon crystal, offering higher efficiency and a uniform appearance.
- Polycrystalline (Multi-Si) Cells: These are comprised of multiple silicon crystals; they are less efficient but more cost-effective to produce. In 2016, multi-Si panels dominated the market with a 55% share, while mono-Si panels held 45%.

In silicon-based photovoltaics, p-type doped silicon ingots are utilized [32] and sliced into thin wafers up to 1/3 mm thick. These ingots can be polycrystalline with a square cross-section or monocrystalline, which are shaped into squares before further processing. To enhance light absorption, wafers undergo texturing through etching, followed by phosphorus doping to create a p–n junction. An anti-reflective nitride coating is then applied, resulting in the cells’ distinctive dark blue appearance. Silver metallization paste is screen-printed on both sides, and the process concludes with sintering to establish a conductive connection between the metallization and silicon.

**Figure 2:**
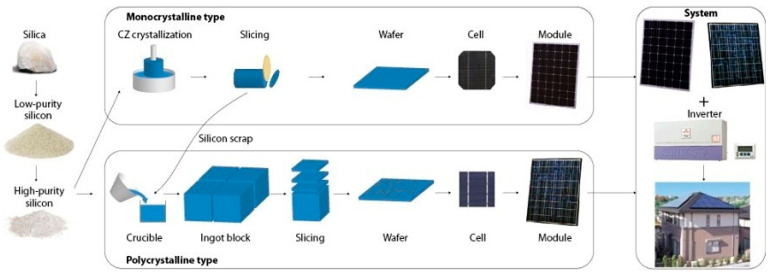
The production process of photovoltaic panels [23].

Thin-film technology, in contrast, involves depositing layers of amorphous or microcrystalline silicon onto substrates like glass or fabric (Figure 2). This approach reduces material usage and production costs but yields cells with lower efficiency and a shorter lifespan [19].

Solar panels at the end of their life (EOL) can become a source of hazardous waste, despite the significant benefits of solar energy development. The global installed capacity of photovoltaics was approximately 400 GW at the end of 2017, and forecasts indicate an increase to 4500 GW by 2050. In Europe, the ban on waste export promotes the recycling of photovoltaic components, in line with the EU policy prioritizing the recovery and recycling of materials from photovoltaic waste [7].

Significant technological advancements in renewable solar energy systems have been occurring since 2022 and include modern cell technologies aimed at providing better absorption parameters while maintaining economic viability. Among these are tandem cells, perovskite cells, potentially bifacial solar panels, and integrated transparent solar cells [33]. These increasingly popular technologies boast greater efficiency compared to older technologies due to the development of flexible, lightweight panels and the introduction of advanced structures and nanomaterials for solar energy collection. Technological progress has also been spurred by the introduction of innovative, optimized production techniques utilizing artificial intelligence and roll-to-roll printing [34].

In 2020, silicon solar cells achieved an efficiency of 26.7%, which turned out to be the maximum efficiency level attainable with this technology. Therefore, further research focuses on the potential for reducing production costs and improving stability [35]. For thin-film cells, the efficiency reached 23.4%. Current scientific research is focused on increasing stability in electricity generation and searching for innovative materials that can be used to develop modern cell production technologies [36,37].

Technological trends are constantly evolving, as new issues related to the development of photovoltaic (PV) technologies are increasingly emerging. Additionally, there is a growing interest in integrating PV technologies into hybrid systems, which ensure stability in electricity generation. This, in turn, leads to increased energy independence for the area [38,39].

## 4. Assessment of Photovoltaic Waste Management and Recycling Practices

**Figure 3:**
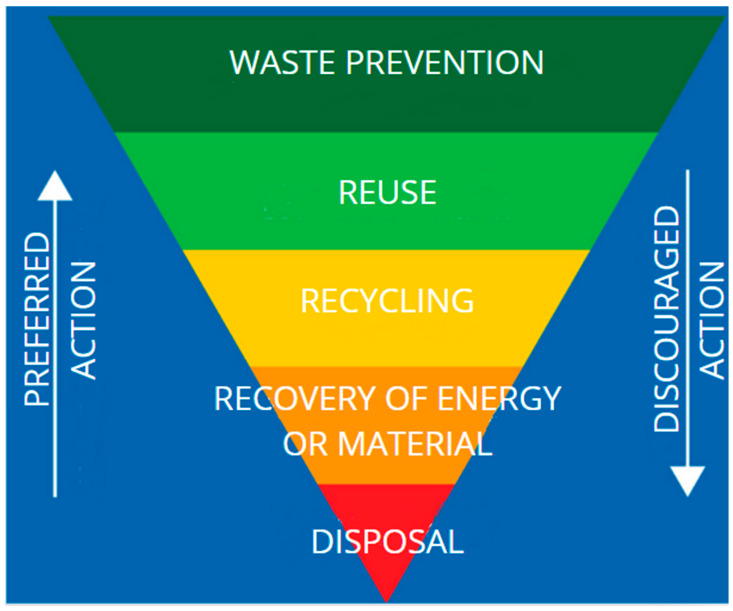
Closed-loop waste management pyramid [41].

With the increasing use of photovoltaic technology, concerns about the environmental impact associated with the approaching end of the lifespan of photovoltaic cells are growing. The popularity of solar energy systems is rising rapidly, as evidenced by the increase in electricity production by these systems from 40,336 kW in 2010 to 942 GW in 2021 [40]. The increased installed systems is associated with a large amount of post-use waste that must be managed shortly. The number of used cells is expected to continue to grow, which is why the European Union and local governments are starting to emphasize the development of appropriate waste identification and management processes. The different technical properties of materials used in photovoltaic panels necessitate proper handling of the resulting waste. The complexity of photovoltaic waste requires the preliminary separation of layers, as discussed in Section 2 above. According to EU regulations, the waste management hierarchy envisages a series of processes that aim to manage the resulting waste rationally and in the least environmentally invasive manner. Many countries in the European Union have to introduce standardized waste handling procedures. For example, in Poland, the National Center for Climate Change serves as the primary institution focused on mitigating climate change, concentrating on strategies to reduce waste generation. This institution has presented the results of its work in the form of a hierarchy for managing decommissioned products, illustrated in Figure 3, which delineates the division into individual categories.

**Figure 4:**
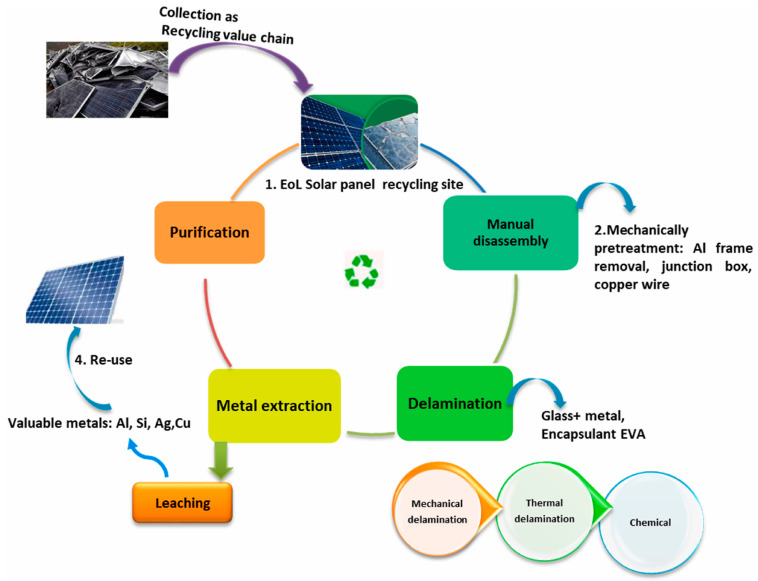
Methods of the utilization of PV cells [42].

The growing issue of photovoltaic panel waste management requires the implementation of a sustainable management system that enables effective recovery, recycling, and reuse of their components. The recycling process of panels itself is complex and requires significant financial investment. Efficient management of decommissioned panels is crucial for the sustainable development of the renewable energy sector. The primary methods for minimizing the environmental impact of this waste include mechanical, thermal, and chemical recycling, as well as the reuse of components. The main methods of PV cell utilization are presented in Figure 4.

The recycling processes of photovoltaic panels vary depending on the module structure, such as between silicon-based (c-Si) and thin-film panels. For silicon-based modules, the goal is to separate the glass and recover silicon cells and other metals. Recycling silicon-based panels requires the removal of the encapsulant, which can be achieved using mechanical, thermal, and chemical processes [7]. As a PV module generally speaking consists of about 70% glass, 10% adhesive sealant, 10% aluminum, 5% silicon, and 5% other metals, including the all-important silver, tempered glass, and aluminum frames, silver is the most expensive component with a large carbon footprint from its original production. This translates into its great importance as an economic factor for panels, as a massive shortage of this element is forecast by 2075 [12].

Still, most of the current methods used to process PV waste are at the laboratory level, which translates into great difficulties in implementing a suitable method on a large scale. Laboratory studies aim to assess the feasibility of recycling photovoltaic panels, including material purity, cost, and potential performance issues. At this stage, no standardization of procedures can be developed. Due to the very high glass content of the panel structure, it is worth considering the inclusion of cullet in cements.

### 4.1. Mechanical Recycling

One of the primary methods is mechanical recycling, which involves the physical dismantling of panels and the recovery of valuable materials such as glass, aluminum, copper, and silicon. This process is relatively simple and inexpensive, although it may not ensure the highest purity of recovered materials. The stages of mechanical recycling include dismantling, crushing, and the selection and separation of components [43]. In the first stage, panels are disassembled, and aluminum frames and electronic components are removed, allowing for their reprocessing. To minimize the use of human forces during disassembly, separating the frame and the remaining electrical infrastructure, Mahmoundi et al. used a mechanical arm in combination with an automated Cartesian robot during the study [42,44]. Then, the panel is mechanically crushed into smaller pieces [45,46], and different materials are separated using methods such as magnetic separation (separating ferromagnetic metals), electrostatic separation (utilizing differences in electrical conductivity of materials), and gravitational separation (distinguishing materials of different densities) [2,45,47,48,49,50,51,52,53,54]. The frame, which provides mechanical strength to the panel, can be recovered through secondary metallurgy after separation [45]. Finally, optical sorting techniques are used to separate glass from other components, and the recovered glass is cleaned, allowing for its reuse.

Mechanical recycling has numerous advantages, including low operational costs and simplicity, as it does not require complex chemicals or high temperatures. Additionally, this process allows for the efficient recovery of aluminum and glass, which make up a significant portion of photovoltaic panels and can be reused. However, mechanical recycling also has certain limitations. Specifically, the recycled material often exhibits lower purity, which restricts its potential for reuse in the production of high-quality products. Some mechanical methods may not ensure complete separation of all components, leading to the loss of valuable materials, and the silicon itself may be damaged during the crushing process, further limiting its value.

The future of mechanical recycling of photovoltaic panels focuses on improving the efficiency of separation and purity of recovered materials [55]. The development of advanced optical sorting systems and micro-separation technologies can enhance the efficiency of this process. The increase in photovoltaic panel waste in the coming decades underscores the importance of developing and optimizing recycling processes to minimize environmental impact. Despite certain limitations, mechanical recycling will continue to play a significant role in managing waste from the photovoltaic sector.

While investigating mechanical methods, Sim et al. designed a special method for separating photovoltaic waste by crushing and screening, which allowed more of the coarser fraction polymers to be retained, while the finer fraction contained metals that were valuable for recovery. The possibility of using a CNC-controlled conventional machine was also investigated [12,56]. The method studied by Mukwevho aimed to grind by cutting with a jet of water, which allowed pure intact glass to be retained while removing the silicon layer along with the EVA [57]. Deng et al. used a mechanical separation technique with very high recovery of cullet and a mixture of Si powder as well as metal [58].

Chen et al. [43] conducted a comprehensive comparative analysis of mechanical recycling methods for silicon photovoltaic cells, presented in Table 1 and Table 2 below.

### 4.2. Thermal Recycling

Thermal processes in the recycling of photovoltaic panels involve the use of high temperatures to remove polymer components, such as EVA film, and the recovery of metals and glass [43]. Removal of the vinyl acetate and ethylene acetate copolymer layer that forms the adhesive layer is proving to be crucial [62,63]. This technique is effective because it allows high-purity materials to be obtained, but it requires advanced infrastructure and generates high energy costs and emissions [64].

Thermal recycling is highly efficient in recovering high-purity materials, but is also expensive due to its energy intensity. The process uses high temperatures to separate and process the materials that make up the photovoltaic panels. The two main stages of the process involve placing the panels in furnaces where temperatures can reach several hundred degrees Celsius. The high temperature burns the plastic and organic layers, facilitating the separation of metals and silicon. Plasma combustion is an advanced method in which a very high-temperature plasma allows the materials to be broken down into their constituent parts, allowing pure metal and silicon to be obtained and toxic substances to be eliminated.

Thermal recycling of photovoltaic panels is effective in obtaining high-purity materials, but its main limitations are the high energy cost and the potential emission of pollutants.

Pyrolysis, a process of material degradation occurring anaerobically at high temperatures, is considered one of the most effective methods of thermal recycling [43]. The anaerobic nature of the process positively influences the prevention of harmful dioxin formation and oxidation [65]. In the case of photovoltaic panels, which contain various materials mixed with plastics, pyrolysis involves the breakdown of organic elements into gases and liquids, leaving metals and glass practically intact [63]. Research on pyrolytic treatment has shown that 99% of polymers in PV modules can be safely removed [66]. The separation of EVA allows for the recovery of semiconductors with nearly 100% purity [9], while the adhesive layer typically undergoes carbonization. Chen et al. [43] developed a comprehensive review of thermal treatment methodologies, presented in Table 3 below, showcasing different approaches to thermal recycling. The goal is to recover intact PV cells while simultaneously removing the EVA coating.

### 4.3. Chemical Recycling

Chemical recycling of photovoltaic panels involves the use of chemicals in solution form, which dissolve the individual layers of the panels, allowing for the separation of valuable metals such as silver and copper. This method enables the recovery of high-purity materials, but it is costly and requires precise control of chemical processes and safeguards against environmental contamination. The advantages of chemical recycling include high efficiency in recovering metals and semiconductors, while the disadvantages include high operational costs and the necessity of using strong chemical agents [67]. The chemical recycling process can be divided into several stages, including dissolution, precipitation, purification, and filtration. Panels are placed in chemical solutions, such as hydrofluoric acid, to dissolve silicon, metals, and glass. After dissolution, various materials are precipitated from the solution using appropriate reagents. The precipitated materials are then purified and filtered, allowing for the recovery of pure fractions of metals, silicon, and other components [68]. Chemical recycling of photovoltaic panels is a key strategy in the circular economy, helping to reduce the environmental impact of waste and maximize resource utilization. Hydrometallurgical and pyrometallurgical processes enable the recovery of pure materials such as silver and copper using solvents and chemical reactions. The main challenges are the costs and potential environmental impact if non-eco-friendly solvents are used [69]. To achieve high-purity silicon, chemical recycling methods are an integral part of the recovery process of purified materials. This method is used not only to recover silicon but also other valuable metals, optimizing resource utilization [63,70,71].

Due to the high economic and technological value of silver, interest in its recovery from PV installations has increased. Consequently, research has begun on the use of leaching technology with nitric acid. The extraction method has been enhanced by the use of electrolysis technology [11,72,73,74], which has gained popularity in recent years. Unfortunately, extraction with this solution proved to be environmentally hazardous due to the emission of harmful fumes [75,76]. Numerous experiments have long been conducted to facilitate silver recovery through leaching, involving the immersion of silver-containing waste in H_2_SO_4_, followed by HNO_3_. To identify the silver content, X-ray fluorescence analysis was used [75].

In a study comparing chemical recycling methods, Min et al. found that the use of ethylene glycol diacetate (EGDA) was the most effective of the selected solvents tested, as EGDA showed the ability to penetrate the narrow crevices of EVA and glass layers [77]. Xu et al. presented a cell etching process using HCl and perchloric acid [78].

To address the issues related to the emission of toxic fumes during leaching, researchers began using a mixture of methanesulfonic acid with an oxidant, which enables the oxidation of silver from the modules [79]. The application of this method leads to increased solubility of metal salts [70]. Chemical processes are considered among the most promising methods for recovering precious metals, which is why Chen et al. [43] provided a summary of chemical methods, which is described in Table 4, Table 5 and Table 6 below, illustrating the careful application of methods following sustainable environmental development.

Research into the end-of-life recovery of silicon wafers from PV modules indicates the effectiveness of other new methods, such as the use of etching paste and HNO_3_ and KOH solutions to dissolve metal Ag and Al electrodes. This environmentally friendly method minimizes water consumption and eliminates the need for harmful surfactants. The thickness of the recycled wafers is above 180 μm, which is suitable for current production processes [2]. It is important to note that the use of nitric acid (HNO_3_) and potassium hydroxide (KOH) solutions in the disposal of photovoltaic panels can pose environmental risks if not managed responsibly. Such processes are mainly used to treat glass and metals but carry the risk of chemicals. HNO_3_ is used to etch metals and dissolve protective layers to recover valuable materials such as silver or silicon, while KOH is used to remove silicon layers or as a component of chemical processes involved in material separation. To avoid risks to the environment, waste must be neutralized. Nitric acid can be neutralized with sodium hydroxide (NaOH), while potassium hydroxide can be neutralized with sulfuric acid (H_2_SO_4_) or another acid. It is also necessary to process chemical waste in special installations that remove heavy metals and other harmful substances and to control vapor emissions by using appropriate closed systems equipped with filters. Applying so many safeguards to chemical processes affects their economy.

### 4.4. Reuse

Another approach to managing decommissioned photovoltaic panels is their reuse, known as re-purposing. This involves using panels that can still generate energy, despite their reduced efficiency, in less demanding applications. Examples of such applications include powering low-power systems such as street lighting, agricultural irrigation systems, or portable devices [70]. These panels can also be used in educational and demonstration projects, which play a key role in educating future researchers and professionals in the field of renewable energy sources.

### 4.5. Landfilling

Landfilling remains the most widely used waste management method in the world. Its popularity is due to its simplicity, low cost, and the lack of dedicated disposal facilities for many types of waste, which further encourages its widespread use. This is also true for photovoltaic panels, which are most economically deposited in landfills, but their negative environmental impact motivates society to find the best method of recycling.

Landfilling is a method that involves transporting end-of-life panels from the place where they were installed and used to a landfill. They are stored whole without the use of additional mechanical, chemical, or thermal methods. Leaving them whole is supposed to favor the prevention of toxic substances escaping from them. However, the panels, like any other object and material, are subject to deformation and damage, so there is still a risk of environmental pollution.

Photovoltaic panels, especially those made of crystalline silicon and thin-film panels, contain toxic materials in their composition, such as lead, cadmium, selenium, antimony, and nickel. They also contain chromium and copper. These materials can pose a risk, especially in the case of uncontrolled storage. The development of modern PV technologies such as perovskite cells introduces additional materials (mainly lead in most current versions of these cells) with negative environmental impacts [83].

### 4.6. Full Recycling Procedure

The sustainability of the photovoltaic industry requires the pursuit of a closed-loop economy, which reduces the use of energy and materials throughout a product’s life cycle, thereby reducing environmental impact.

The full recovery end-of-life photovoltaic (FRELP) procedure was implemented after the Waste of Electrical and Electronic Equipment (WEEE) Directive [84] was passed by the European Union, which introduced the principle of extended liability imposed on photovoltaic panel manufacturers and the need to bear the costs of dismantling, managing, and monitoring waste. FRELP combines chemical and mechanical processes (manual or machine dismantling of the frame and cables, glass separation and refining, incineration, fly ash screening, acid leaching, filtration, and electrolysis) to separate and recover most of the materials that make up the panel. Improvements in this technology have further introduced sequential electrowinning to produce SiNx, which is then subjected to chemical etching [85].

Further work on the development of more efficient and sustainable recycling methods for PV panels is necessary to create an efficient model for the management of this waste based on circular economy principles.

### 4.7. Utilization and Recycling of Photovoltaic Panels

At the end of the service life of solar thermal installations, which generate electricity by converting solar projects, there are opportunities to recycle, as well as revitalize, the components included in the construction of said installations, which become waste in their entirety. Due to the fairly specific construction of photovoltaic panels, which are made of many materials that are not typical for recycling, they require appropriate and specialized treatment through the use of technologies that are not yet so popular and require further development.

Both disposal and recycling are used for the treatment of end-of-life photovoltaic panels. The treatment procedure involves dismantling the panel into individual parts, which are then recycled or disposed of in an environmentally friendly manner. Recycling is carried out when it is technically feasible and economically viable, while the remaining components are disposed of. The largest component by mass in a photovoltaic panel is the cover glass, which constitutes approximately 63% of the total weight, followed by the aluminum frame at around 22%. Both materials can be fully recovered, with the cover glass achieving nearly 100% recyclability in most cases. In contrast, the plastics used for encapsulation are difficult to recycle. The panels also contain heavy metals such as lead, silver, tin, and copper, which are essential due to their toxicity, but their recycling is difficult and expensive, comparable to the extraction of new raw materials [86,87].

### 4.8. Emerging Trends in Sustainable Recycling Practice

The requirement to minimize the impact of waste generated by the renewable energy sector underlines the need for the dynamic development of technologies to reduce environmental degradation. The outlook in the area of recycling points to the need for advanced action in two key directions: the development of alternative, greener materials for photovoltaic panels and the implementation of modern recovery technologies. Of particular importance are innovations, such as electrochemical methods, which offer the possibility of significantly improving the efficiency of the recycling process through precise and efficient recovery of critical raw materials.

Such solutions contribute to closing the life cycle of materials while increasing their use value in subsequent industrial processes, in line with the circular economy [88].

Each of the above-mentioned recycling methods has its strengths and weaknesses. The mechanical method is relatively simple and cheap but does not provide a high purity rate of the recovered materials. Thermal methods allow the recovery of cleaner materials but with a high energy and financial investment. Chemical methods allow the recovery of clean materials but require an appropriate infrastructure and strict safety standards. The introduction of new technologies will create new opportunities for the industry, potentially increasing the efficiency and reducing the costs of recovery processes.

More and more promising options for waste management are emerging on the market, including laser [89] and biotechnological recycling technologies. Laser technologies make it possible to obtain the silicon base, after precise separation and removal of unwanted layers (anti-reflective layer, metallization, and p–n semiconductor) in a purification process [90], while the biotechnological method is based on the participation of micro-organisms to achieve the biodegradation of organic panel components.

## 5. Utilization of Waste Glass in Cement Production: Assessment of Innovative Strategies

### 5.1. Waste Glass from Recycled Photovoltaic Panels

The amount of recovered glass depends on the type of panels. First-generation panels, which include silicon panels, contain about 65–75% glass by total weight, so there is about 7–11 kg of glass per 1 m^2^ surface area. Second-generation panels, which include thin-film panels, have a much thinner layer of glass than the first generation, but the proportion of glass by total weight is about 10–13 kg of glass per 1 m^2^ surface area, which is almost 90%. For third-generation panels, which include perovskite and organic panels, the proportion of glass is only 2–8 kg per 1 m^2^.

Glass waste from photovoltaic panels, in the context of obtaining secondary raw materials, can be of key importance for the construction industry, in the production of Portland cement. 

Such an additive can perform several important functions, such as:

- Significantly improving the quality, strength, and structural stability of cement, as well as its mechanical properties;
- Reducing the extraction and consumption of natural aggregates such as sand and gravel;
- Beneficially impacting the environment by reducing its degradation;
- Increasing resistance to chemical corrosion, resulting in higher durability of building structures under varying environmental conditions;
- Reducing waste volumes;
- Supporting the objectives of sustainability legislation.

The above possibilities underline the need for further research into using a cullet as an additive in cement mortar, given the perceived potential to improve environmental quality and significantly influence sustainable construction development.

### 5.2. Cullet in Cement Production

A study by Fernández et al. [91] analyzed the feasibility of recycling photovoltaic panels for cement production, focusing on integrating silicon cells into cement systems. The recovery process includes steps such as rotor and hammer crushing, thermal treatment, and screening, allowing up to 85% of the mass of the panels to be recovered as waste glass of specific fractions [45]. This method is in line with Directive 2012/19/EU of the European Parliament and of the Council of 4 July 2012 [84], concerning waste electrical and electronic equipment.

Currently, no standardized guidelines specifically regulate the incorporation of photovoltaic (PV) waste into concrete mix designs exist. However, research has explored the feasibility of using materials recovered from end-of-life PV panels, particularly glass, as partial replacements for traditional concrete components.

Studies have investigated subsites with crushed solar panel glass in concrete production. For instance, one experimental investigation evaluated the use of solar panel waste as a sand replacement in concrete, examining its effects on the material’s properties [92]. Another study focused on using glass from decommissioned PV panels in cement composites, analyzing the impact on mechanical and durability characteristics [93].

While these studies provide valuable insights, the absence of formal guidelines means that implementing PV waste in concrete requires careful consideration of factors such as particle size distribution, potential chemical reactions (e.g., alkali–silica reaction), and the overall influence on concrete performance. Until standardized regulations are established, such applications should be approached on a case-by-case basis, guided by thorough research and testing to ensure structural integrity and safety.

According to numerous studies, climate neutrality regarding greenhouse gas emissions is forecast to be achieved in Europe by 2050 [94,95]. In recent years, there has been a growing interest in cements with reduced Portland clinker content as a response to the need for sustainability in the construction industry. These types of cements are gaining popularity not only due to environmental aspects, such as the reduction of carbon dioxide emissions associated with clinker production but also due to the increasingly well-understood properties of mineral additives. Such additives include granulated blast furnace slag, silica dust, fly ash, or lime [96], which allow the properties of cement to be modified, making it more versatile and resistant to various degradation factors.

Multi-component types of cement containing these additives now account for more than half of the total cement production in many developed countries [97]. This trend is due to the numerous advantages brought by mineral additives. Granulated blast furnace slag and silica dust, which are among the most commonly used additives, have been extensively studied for their effects on the microstructure and properties of cementitious materials. The technical and scientific literature [98,99] emphasizes their positive effects, especially in terms of the durability of the finished products. These additives have been shown to increase the density of the cement microstructure, reducing its permeability and limiting its calcium hydroxide content—a compound susceptible to corrosion. As a result, multi-component types of cement have better resistance to aggressive chemical environments, including acids, sulfates, and salts.

However, despite the many benefits of mineral additives, their availability is becoming increasingly limited. For example, the closure of many steel mills has contributed to a significant decline in the production of granulated slag, a byproduct of the blast furnace process [100,101,102]. This situation is forcing the cement industry to look for alternative raw materials and additives to replace the traditionally used components.

One of the potential solutions to this problem is to use waste glass as an additive for cement. Cullet, a byproduct of glass recycling, contains significant amounts of reactive silica, making it a promising substitute for traditional mineral additives. The chemical composition of waste glass varies widely and depends on its original use and source [103]. In an alkaline environment, typical of cement slurries, ground waste glass can react with non-crystalline silica contained in aggregates [104]. This process, known as the alkali –silica reaction, leads to the formation of an expansive gel, which causes microcracks in the cement structure and significantly reduces its mechanical strength [105].

Concerns about the alkali–silica reaction limited the use of waste glass in cement and concrete technology for many years. It was widely believed that its use carried a high risk of expansion, which made this material unattractive [106]. However, more recent studies have shown that appropriate grinding of waste glass makes it possible to reduce the risks associated with this reaction. With a sufficiently high degree of grinding, the glass begins to exhibit pozzolanic (binding) properties [104], i.e., the ability to react with calcium hydroxide in the cement matrix in the presence of water, leading to the formation of permanent chemical compounds that strengthen the concrete structure.

Among the materials most commonly used in concrete technology are fly ash, silica dust, and metakaolin, which have long been recognized for their chemical and pozzolanic properties. However, glass cullet, especially from photovoltaic waste, is attracting increasing attention, offering a wide range of advantages over traditional additives, both in terms of availability and environmental co-benefits.

Fly ash has been a valued mineral additive for years, known for its ability to improve the microstructure of concrete, reduce permeability, and increase resistance to chemical corrosion. However, its availability is becoming an increasing problem due to the energy transition and the shift away from coal. Declining production of this raw material is leading to an increase in its price and a reduction in its wide applicability. Compared to fly ash, cullet is characterized by greater availability, as glass waste, especially those difficult to process chemically, is produced on a global scale. In addition, glass cullet is characterized by a more stable chemical composition, which eliminates the problem of variability in properties that occurs with ash from different sources.

Metakaolin, which is also an active additive, is distinguished by its very high pozzolanic activity, which allows it to significantly increase the durability of concrete. However, its production is energy-intensive and costly, which limits its availability and use in large-scale projects. Glass cullet, which is a secondary waste, is much more environmentally friendly and economical. Although its pozzolanic properties are not as intense as those of metakaolin, when properly ground, cullet offers comparable benefits in terms of improved durability and resistance to aggressive environments.

Silica dust, considered one of the most effective pozzolanic additives, is characterized by its ability to significantly reduce permeability and increase the strength of concrete. However, its limited supply and high cost mean that its use is reserved mainly for projects with high technical requirements. Glass cullet, although less pozzolanic active, due to its availability and lower cost, can be used on a much larger scale, making it a more versatile solution. Importantly, proper grinding of glass cullet allows activation of its pozzolanic properties, which are similar to those offered by traditional mineral additives. Fine cullet (grains of less than 75 µm) can be used as an additive to Portland cement, leading to a reduction in CO_2_ emissions, improved mechanical properties of concrete, and increased corrosion resistance. Studies show that the long-term strength of concretes with cullet additives can exceed that obtained with fly ash or silica dust, confirming its utility value.

Environmentally, cullet stands out among the materials in question. It is a waste that largely ends up in landfills, so its use in concrete technology fits into the concept of a closed-cycle economy. The use of this raw material makes it possible not only to reduce the amount of waste, but also to reduce the consumption of natural resources and reduce carbon dioxide emissions. This makes cullet a material that meets both the technical requirements and needs of the modern construction industry. Glass cullet derived from photovoltaic waste is an innovative and competitive solution compared to traditional pozzolanic additives such as fly ash, metakaolin, or silica dust. Its availability, stability of chemical composition, pozzolanic properties, and environmental benefits make it an attractive alternative in concrete mix design. The large-scale introduction of this raw material into industrial practice has the potential to create more sustainable, durable, and economical solutions that address today’s environmental challenges.

The determination of the critical glass grain size at which alkali–silica reactions are inhibited is not clear. Research results indicate that a safe grain size is usually below 0.35 mm [104], but some studies suggest that larger sizes, up to 1 mm, are also acceptable. At the same time, it has been proven that the smaller the glass grains are, the greater their pozzolanic activity, making them a more effective addition to cement. For example, ground glass powder with a specific surface area in the range 0–0.079 mm has been shown to have an effect similar to fly ash [107] or silica dust [108], reducing the shrinkage of mortars and concretes and improving corrosion resistance, including chloride resistance.

In terms of performance, ground glass can be compared to fly ash. The introduction of glass as an additive to cement leads to changes in the consistency of the concrete mixture and a reduction in its early compressive strength. However, over longer hydration periods, the strength of mixtures with glass additives usually exceeds the values achieved by reference samples without additives [109]. Studies show that the increase in strength varies from 7% to as much as 67%, depending on the glass content, the type of aggregate used, and the maturation conditions of the sample. Furthermore, the hydration process can be accelerated by using higher temperatures during curing.

On the other hand, high concentrations of glass in the mix may lead to some limitations. For example, cements in which glass replaces more than 10% of the Portland clinker by weight [110] may experience a decrease in strength over longer curing periods. A study by Shayan showed that concrete with 20% glass content in the cement matrix after 90 days showed a strength 18% [109,110] lower than a control sample. However, after one year, the strength increased by 10% over the reference sample, reaching 70 MPa. In the case of cement in which glass constituted 30% by weight, the strength after one year was only 40 MPa, a significant decrease compared to the control sample.

In conclusion, ground waste glass has the potential to become a valuable addition to cement, especially in the context of the limited availability of traditional mineral additives. However, the successful use of this material requires precise control of its physical and chemical properties, including the appropriate degree of fineness, to ensure the durability and strength of the final concrete products.

### 5.3. Use of Photovoltaic Panel Elements in the Cement and Concrete Industry

The cement and concrete industry, given the increasing amount of post-operational waste from solar installations, is becoming the only one of the key opportunities for processing and reuse. Tempered glass, silicon, and polymers from photovoltaic panels can find use as secondary raw materials in production processes. In line with the construction of photovoltaic modules, tempered glass, which accounts for about 70 percent of the weight of photovoltaic panels, is one of the most important components for potential use. In cement plants, glass, once properly crushed and cleaned of polymer layers and me-tools, can be used as a flux in cement kilns.

The introduction of toughened glass reduces the firing temperature of the clinker, which in turn reduces energy consumption and CO_2_ emissions. Thanks to its high silica content, the glass also promotes the formation of the chemical structure of the clinker. In the production of concrete, tempered glass, ground to a suitable granularity, can be used as a filler. This additive increases the density of the concrete mixture and improves its mechanical properties, such as compressive strength. In addition, it improves the durability of the concrete and its resistance to aggressive chemical agents [111,112].

Silicon, an essential ingredient in photovoltaic cells, can be ground and used as a pozzolanic additive in Portland cement after purification from heavy metals and polymer layers. In this role, silicon reacts with calcium hydroxide, improving the microstructure of the cement and increasing the durability of the concrete. This additive is particularly useful in structures exposed to aggressive environments, such as water infrastructure or industrial facilities. Silicon from PV panels can also act as a filler in high-strength concretes, improving their mechanical performance. However, this process requires thorough research into the effects of silicon on the properties of the final products, such as flexural strength or resistance to temperature changes [113].

Polymers present in PV panels, such as ethylene vinyl acetate (EVA) and fluoropolymer back-coatings, can be used as an alternative fuel in cement kilns. The process of burning these materials at high temperatures provides the energy needed to produce clinker, while reducing the need for traditional fossil fuels such as coal. To avoid the emission of toxic gases such as hydrogen fluoride, advanced flue gas cleaning systems are required [114].

Copper indium selenide (CIS) photovoltaic modules also contain amorphous silica, which can be used as a mineral additive in concrete mixtures to improve the mechanical properties and durability of concrete. A study of CIS waste showed that its use as a fine aggregate in concrete mixtures, in amounts ranging from 5% to 40%, had a beneficial effect on the density, durability, and mechanical properties of concrete. In the analysis, sand was substituted for CIS PV module waste, which was crushed to particle sizes of less than 4 mm using a jaw mill. CEM I 42.5 R cement, coarse-grained aggregate, water, and plasticizing admixtures were used to produce the samples [115].

The results showed that the addition of CIS waste affected the consistency of the concrete mix, improving it when 5–20% sand was replaced. Glass, which does not absorb water, increases the amount of available water in the mix, improving its workability. The density of the concrete increased specifically at a proportion of 30% waste, as a result of better pore filling by the fine glass particles. At proportions above 40%, however, the density decreased [115,116].

The highest compressive strength (39.3 MPa) was achieved when replacing 5% of the sand with CIS waste, an increase of 17% compared to the control concrete. The pozzolanic reaction of amorphous silica with calcium hydroxide created a more compact concrete microstructure, although strength began to decrease at proportions above 20%. Porosity and water absorption were also reduced, and the CIS waste effectively immobilized heavy metals such as cadmium and copper, reducing their leaching to levels below detection [116].

Leaching tests using the toxicity characteristic leaching procedure (TCLP) and NEN 7375 (Leaching Characteristics—Determination of the Leaching of Inorganic Components from Moulded or Monolithic Materials with the Tank Leaching Test) methods confirmed the effectiveness of immobilization of toxic substances in concrete containing CIS waste. The lowest porosity and water absorption were recorded in concrete with 5% CIS waste, indicating the potential of these materials in structures exposed to water. The only exception was sodium, whose highest leaching was associated with high Na_2_O content in the glass of the CIS modules.

In addition, photovoltaic panels contain metals such as silver, copper, and aluminum, which, when recovered, can be used as micro-fillers in concrete. Aluminum and copper can increase the durability and improve the mechanical properties of concrete. However, the recovery process for these metals is more complicated and costly, requiring further research into the cost-effectiveness of their use [115,116]. Despite the many advantages of using PV panel components in the cement and concrete industry, there are several technological challenges. Processes such as grinding, segregation, and purification of materials are costly and time-consuming. Some elements, such as lead or fluorine, can pose an environmental risk if not properly removed. It is also necessary to adapt existing production processes in cement and concrete plants to the new secondary raw materials. The use of photovoltaic panels in the cement and concrete industry is a promising direction with the potential to significantly reduce waste and reduce the consumption of primary raw materials. However, it is crucial for technologies that allow efficient glass, silicon, and polymers processing, while eliminating pollution and minimizing costs [117].

## 6. Future Prospects

The incorporation of glass waste from photovoltaic (PV) panels into cementitious materials presents a promising avenue for sustainable construction practices. As the demand for renewable energy sources continues to rise, the volume of PV panel waste is expected to increase significantly. Therefore, developing effective recycling strategies for this waste is crucial.

1. Research and Development: Future research should focus on optimizing the processing techniques for glass waste to enhance its properties as a cement additive. Investigating various particle sizes, shapes, and compositions can lead to improved performance in concrete mixtures. Additionally, studies should explore the long-term durability and environmental impact of using glass waste in cement.
2. Technological Innovations: Advancements in recycling technologies are essential to streamline the extraction and processing of glass from end-of-life PV panels. Innovations such as automated sorting systems and eco-friendly solvents can facilitate the recycling process, making it more efficient and cost-effective.
3. Regulatory Frameworks: Establishing supportive regulations and standards for the use of recycled materials in construction will be vital. Policymakers should encourage the integration of recycled glass in cement production through incentives and guidelines that promote sustainable practices.
4. Industry Collaboration: Collaboration among the renewable energy sector, construction industry, and waste management organizations can foster a circular economy. By working together, stakeholders can develop comprehensive strategies for the collection, processing, and utilization of PV glass waste, ensuring that it contributes positively to both environmental sustainability and economic growth.
5. Public Awareness and Education: Raising awareness about the benefits of using recycled materials in construction is crucial. Educational initiatives can inform builders, architects, and the general public about the advantages of incorporating glass waste into cement, promoting a shift towards more sustainable building practices.

In conclusion, the future of incorporating glass waste from PV panels into cementitious materials is bright, with significant potential for enhancing sustainability in the construction industry. Continued research, technological advancements, regulatory support, and collaborative efforts will be key to realizing this potential and addressing the challenges posed by increasing PV waste.

## Figures and Tables

**Table 1:** Analysis of mechanical recycling methods for PV: Part 1 [43,47,59,60].

Method	High-Voltage Fragmentation	Cement Matrices	Thermal Treatment	Electro-Hydraulic Fragmentation
Use	Separation and recovery materials	Recycling materials	Glass recovery	Metals recovery
Recovery Rate	-	-	+/−85%	99% Cu, 60% Ag, 80% Pb/Sn/Al
Recovered Materials	Glass, Cu, Sn, Pb, Ag	-	Glass	Si, Ag, Cu, Sn, Pb, Al
Challenges	Improved recovery	Less mechanical strength, high porosity	Emissions management	-
Impact	Commercial viability	-	Reduces consumption of energy and chemical	Economical
Advantages	Separation and recovery of materials	Create insulation and soundproofing materials	High glass recovery rate	Easy metal recovery
Disadvantages	Need to use another method for recovery of higher Ag	Does not recover materials for direct reuse	Further processing for metal recovery	-

**Table 2:** Analysis of mechanical recycling methods for PV: Part 2 [43,46,55,59,61].

Method	Triple Crushing	Electrostatic Separation	High-Voltage Fragmentation	Supercritical CO_2_
Use	Recycling	Separation waste wires	Recovery metals	Separation of solar cell from encapsulation and glass layer
Recovery Rate	91%	68.6% Cu with over 100% purity	95% Cu and 96% Ag	>96%
Recovered Materials	Glass, Al, Cu, (Ag)	Cu, Al	Cu, Al, Pb, Ag, Sn	Glass, Pb filaments, back sheet
Impact	Economically feasible	-	-	Use of toluene
Advantages	Less thermal waste	High purity	Various size crushing	Less solvent usage
Disadvantages	-	Need to find new model improvement for Al particles	-	-

**Table 3:** Review of recycling methods based on pyrolysis for removing EVA [9,43,49,50,63,65].

Method	Thermal Treatment and Mechanical Force	Pyrolysis 500 °C	Organic Solvents	Thermal Treatment	Two-Stage Heating
Significant Findings	Obtaining EVA extracted similar properties to commercial	75% of polymers degrade	Silicon recovery of over 86% with high purity	Detection of metals	Integral recovery of TPT backing materials; EVA binder removed
Advantages	Eco-friendly	Over 100% removal of polymers	Efficient silicon recovery	Highlights hazardous metal emissions	Detailed analysis of EVA pyrolysis
Challenges and Limitations	-	Mass loss	-	Need to adequately emissions	Treatment of pyrolysis products
Applications	Reuse of extracted EVA	-	New method of silicon supply and costs	-	Efficient recycling of waste PV panels

**Table 4:** Summary of chemical methods used in recycling silicon photovoltaic panels: Part 1 [11,43,63,80].

Method	Mechanical and Nitric Acid Leaching	Nitric Acid Leaching	Organic Solvent	Organic
Target Material	Ag	Si, Cu, Ag, Pb	Si	Si
Key Process	Leaching in HNO_3_, precipitating use NaCl	5 M nitric acid, agitation at 200 rpm	Trichloroethylene at 80 °C with mechanical pressure	o-Dichlorobenzene at 120 °C
Efficiency	High yield silver concentration	Si: 80%, Cu: 79%, Ag: 90%, Pb: 93%	Recovered without damage after 8 days	Recovered without damage after 1 week
Concerns	Energy consumption	Handling of acids	Swelling and cracking	Cracking EVA

**Table 5:** Summary of chemical methods used in recycling silicon photovoltaic panels: Part 2 [9,43,80].

Method	Solvent Extraction and Electrowinning	Acid Precipitation	Sulfurization and Neutralization Treatment	Chemical Etching
Target Material	Cu	Ag	Pb	Si
Key Process	LIX84-I extraction, H_2_SO_4_ stripping, electrowinning	HCl precipitation, NaOH, hydrazine hydrate reduction, electrolytic refining	NaOH neutralization, Na_2_S sulfurization	HNO_3_, H_2_SO_4_, CH_3_COOH
Efficiency	-	Over 100% purity	Over 93% removal	Yield 86%, over 100% purity
Concerns	Chemicals	Chemicals, high-temperature processes	Toxic compounds	Strong acids

**Table 6:** Summary of chemical methods used in recycling silicon photovoltaic panels: Part 3 [43,48,70,81,82].

Method	Ultrasonic Irradiation	Chemical Refinement	Chemical Refabrication	Chemical Recovery and Electrorefining
Target Material	EVA	Si	Si	Ag
Key Process	O-DCB, TCE, benzene, toluene, ultrasonic power	Thermal or chemical separation followed by chemical refinement	Wet chemical process using a mixture of HNO_3_ and HF	Methanesulfonic acid (MSA) mixed with H_2_O_2_, purification by electrorefining
Efficiency	Complete dissolution	Silicon recovery	Re-fabrication with high efficiency—17.6%	Purity of Ag over 100% after electrorefining
Concerns	Damage in other solvents	Absence of antireflective coating on resultant cells	The optimal ratio of HNO_3_/HF to avoid incomplete etching or deposition of Ag particles	Excessive H_2_O_2_ decomposition and ensuing H_2_O generation

## Data Availability

All data and information are included in the manuscript.
